# Supplementary material for: Second Victim Support at the Core of Severe Adverse Event Investigation
Source: Int J Environ Res Public Health. 2022 Dec 15;19(24):16850. doi: 10.3390/ijerph192416850 (PMC9779208; doi:10.3390/ijerph192416850)
Supplement: Supplementary file 1 [file ijerph-19-16850-s001.zip › ijerph-2052925-supplementary.doc]

**Supplementary Material S1. Summary of the Procedure for Serious Adverse Events (PSAE).** The original document, in Spanish, is available from the authors upon request.

The Procedure for Serious Adverse Events (PSAE) was designed to target healthcare professionals at risk through a comprehensive approach. PSAE is aimed directly at workers who, during their daily activities, may be faced with the need to provide an immediate response to a Serious Adverse Event (SAE).

The first step was to assemble a multidisciplinary group of experts on patient safety. This team systematically reviewed the evidence on action plans for SAE and/or second victims' support programs. The results were translated into an initial proposal that underwent expert consultation including mental health, occupational health, legal counselling, intensive care, surgical and emergency specialists. After integrating expert inputs into the protocol, the final version was approved by the Patient Safety Committee Board and the hospital's governing bodies and disseminated among all hospital workers.

Key features of the PSAE are described below:

1. **Procedure activation.** Any professional who becomes aware of the occurrence of a SAE must proceed to activate the procedure. The worker´s function is to communicate the event to their immediate superior or, failing that, to the center's patient safety referent, either through the center's event notification system or through direct or telephone communication. The NQF List of Serious Reportable Events is used [1].

2. **Immediate actions.** The initial objective is to minimize possible harm to the patient, attend to the professionals involved and protect the prestige of the institution. These immediate actions include:
   a) **Immediate care of the patient involved in the event**. Once the serious or sentinel event has been confirmed, the priority objectives in this phase are to protect the patient from further harm and to carry out those potential actions necessary to stop, reverse or mitigate the harm that has already occurred.
   b) **Immediate support to the workers involved in the event**. The objectives of these actions are to provide emotional first aid and to ensure continuity of care. Unit managers or those professionals who are natural sources of support in the clinical units should act as the first level of support to the healthcare workers involved. These actions correspond to first-level of the ForYOU program [2].
   c) **Activation of the multidisciplinary crisis committee.** The Crisis Committee composition includes trained members of the Patient Safety Committee. This team aims to translate into practice the necessary measures to ensure an adequate response, guarantee correct and timely information to the patient and his/her family, provide adequate care to the affected professionals, and preserve the reputation of the institution and its professionals, beside identity and correct potential associated factors, to avoid the event repetition. The management director and the responsible of the involved clinical units take part only in the first and last meetings of the crisis committee. The activation of the crisis committee should occur within 24 hours since the event is identified. They have to approve the activation of the PSAE and the improvement actions suggested after the root cause analysis.

3. **Management of the SAE.**
   a) **Follow-up care and information to the patient and family**. The objective of the follow-up is to keep the patient and family informed and to provide them with the support they require.
   b) **Internal and external communication plan.** The aim of this plan is to preserve the reputation of the centre and its professionals. Those responsible for internal and external communication are identified. A press release is prepared that summarises the situation in a clear and understandable way, preserving the privacy of patients and professionals and highlighting the centre's response to monitor and improve the care provided. It is only used if necessary. The internal communication plan seeks to avoid speculation and rumours while strengthening the culture of safety.
   c) **Adverse event management.** The assessment of the event must be initiated within 48 hours and completed within 45 days. The crisis committee is split into two groups, one dedicated to professional support and the other to root cause analysis, which will work in a parallel and coordinated manner. Each group will include, if possible, a representative from the clinical unit involved, but not directly involved in the event.

**Attention to all workers involved in an adverse event.** Semi-structured individual interviews are used to assess the impact of the SAE on the workers, regardless of whether or not they have received first-level support. The interview also allows the trained team to identify the signs and symptoms of second victims and offer them the most appropriate approach to achieve individual recovery (equivalent to the second-level of the ForYOU program). Workers receive information regarding professional, psychological, and legal support resources available in the center. Some workers will need a more specialized level of support (equivalent to the Third-level of the ForYOU program) which they can accept or dismiss. If they accept, they are derivate to mental health specialists but if they dismiss it, they still receive a follow-up by the second-level team.

**Analysis of the event.** After the interview, the professionals involved are invited to participate in the analysis of the event. The objective is to identify the immediate causes of the event, but also the underlying modifiable factors related to the organization, resources, training and communication, patients, and individual workers and tasks assigned to prevent future events. Using a system-centred approach, qualitative results from the interviews are collated, analyzed and integrated in the SAE evaluation.

**Final report development.** This report includes identified SAE causes and an improvement plan for the units. The first draft is circulated among the managers of the units involved for their input and a final consensus report is submitted to the hospital board. In this approach, a commitment to the proposed improvement actions is achieved, as they are perceived as their own initiatives.

**4.	Evaluation of the improvement plan.** For the following 12 months, the development, implementation, and impact of the improvement plan are monitored by the PSAE. The initially proposed improvement actions can be adapted or modified through that period if necessary.

References:

1. National Quality Forum (NQF). *Serious Reportable Events in Healthcare—2011 Update: A Consensus Report*; NQF: Washington, DC, USA, 2011.
2. Scott, S.D.; Hirschinger, L.E.; Cox, K.R.; McCoig, M.; Hahn-Cover, K.; Epperly, K.M.; Phillips, E.C.; Hall, L.W. Caring for Our Own: Deploying a Systemwide Second Victim Rapid Response Team. *Jt. Comm. J. Qual. Patient Saf.* **2010**, *36*, 233–240.
